# Editorial: Advances in biomarkers and drug targets: harnessing traditional and AI approaches for novel therapeutic mechanisms

**DOI:** 10.3389/fphar.2026.1885988

**Published:** 2026-06-26

**Authors:** Shaoqiu Chen

**Affiliations:** Department of Geriatric Medicine, John A. Burns School of Medicine, University of Hawaiʻi at Mānoa, Honolulu, HI, United States

**Keywords:** artificial intelligence, biomarkers, drug targets, machine learning, multi-omics integration, precision pharmacology, therapeutic mechanisms, traditional methods

## 1 Introduction

The identification of robust biomarkers and tractable drug targets is the foundation upon which modern precision pharmacology is built. For decades, this enterprise has been driven by hypothesis-led, mechanism-anchored experimentation that combines molecular biology, classical pharmacology, and increasingly powerful omics technologies. These traditional approaches have produced an enduring catalog of clinically meaningful markers and targets—from serum proteins and tissue biomarkers to actionable oncogenic drivers—and continue to deliver the mechanistic depth required for translational confidence (FDA-NIH Biomarker Working Group, 2016). At the same time, the scale, dimensionality, and heterogeneity of contemporary biomedical data have outpaced what conventional analytical pipelines can comfortably accommodate, exposing meaningful gaps in reproducibility, integration across data modalities, and prioritization of candidates for downstream validation.

Over the past 5 years, artificial intelligence (AI), including machine learning (ML) and deep learning, has emerged as a complementary force in this space. AI methods excel at extracting compact signatures from high-dimensional data, integrating heterogeneous evidence layers, and learning representations that can be transferred across diseases (Topol, 2019; Vamathevan et al., 2019). They also enable rapid drug-target and drug-response inference at a scope that hypothesis-driven studies cannot achieve in isolation. Yet AI is not a substitute for biology. Concerns about overfitting in small cohorts, fragile generalization across centers, model opacity, and the gap between *in silico* predictions and clinical utility remain genuine impediments to translation. The most credible path forward is, therefore, neither purely traditional nor purely computational, but iterative: AI prioritizes hypotheses from large datasets; classical experimental and clinical pipelines validate, refine, and contextualize them.

This Research Topic, *Advances in Biomarkers and Drug Targets: Harnessing Traditional and AI Approaches for Novel Therapeutic Mechanisms*, was launched with this iterative vision in mind. We sought contributions that (i) deploy traditional omics or molecular biology to define new biomarkers and targets, (ii) apply AI to extend or accelerate such discoveries, or (iii) explicitly integrate both. The Research Topic that has emerged spans oncology and major chronic non-communicable diseases, includes original research, mini reviews, and reviews, and showcases—rather than catalogs—the methodological logic that is reshaping pharmacological discovery. In this Editorial, we synthesize the principal themes, highlight representative contributions, and outline the open questions that the field must address as it moves from data-rich discovery to clinically robust translation.

## 2 Cancer biomarkers and therapeutic targets: integrating omics with AI

Oncology has been the most active domain in the Research Topic, reflecting both the urgency of unmet clinical needs and the comparative availability of large molecular datasets. Several contributions illustrate complementary modes by which traditional and AI strategies can be combined to refine biomarker discovery, target prioritization, and treatment-response prediction across tumor types.

In non-small cell lung cancer (NSCLC), Qin et al. interrogated the predictive value of mitochondria-derived RNAs (mtRNAs) for chemotherapy response by combining classical machine-learning algorithms with the BiomedGPT framework. Their analysis identified mtRNA features associated with differential chemotherapy benefit and underscored how AI-enabled analytics can extend the utility of biomarker classes that have previously been studied mainly in a diagnostic—rather than predictive—context. The work provides a methodologically explicit example of how generative biomedical models can be wrapped around conventional ML pipelines to refine therapeutic decision-making in NSCLC, while also acknowledging the standard caveats around cohort size and external validation.

Wan et al. focused on thyroid cancer, where the absence of robust molecular biomarkers continues to limit risk stratification and target selection. Their study integrated bulk-transcriptomic meta-analysis with ML-driven feature selection and single-cell RNA-sequencing of more than 50,000 thyroid-cancer cells, then validated key findings in TPC-1 and BHT101 cell lines using miR-6756-5p overexpression and CRISPRi-mediated knockdown, including xenograft experiments. The convergence of these layers yielded a four-feature panel—BID, MIR6756, ITM2A, and TGM2—with strong discriminative performance in training and independent validation sets, and a defined therapeutic axis through miR-6756-5p. This is, in many ways, the prototypical workflow this Research Topic was designed to encourage: AI is used to compress high-dimensional data into a small set of testable candidates, while traditional functional assays establish biological plausibility and mechanism.

Wu et al. address a different but equally important question: how can AI be used to *validate* rather than supplant traditional biomarkers? Focusing on hepatocellular carcinoma (HCC) recovery, the authors review how AI-enabled analyses can be embedded around long-standing markers such as AFP and PIVKA-II, alongside radiomics-derived imaging features, to produce calibrated, interval-specific relapse-risk estimates that support risk-stratified surveillance. They argue—correctly, in our view—that AI-enabled validation in HCC recovery should proceed as a structured pathway that links analytical validity, clinical validity, and clinical utility while preserving the stated intended use. Their treatment of DECIDE-AI for early-stage live evaluation and of SPIRIT-AI/CONSORT-AI for randomized designs offers a usable scaffold for translational rigor.

Finally, in the setting of osteosarcoma, Gao and Wu provide a mini review that articulates an explicit operational definition of “integration”: AI-guided analyses narrow and organize candidates, while traditional experiments provide mechanistic support and functional confirmation, forming an iterative discovery-to-validation loop rather than two parallel evidence streams. The authors deliberately position their contribution as a methodological framework rather than a comprehensive catalog of biomarkers, with selected osteosarcoma examples illustrating how supervised, unsupervised, and network-based ML approaches can interface with traditional pathway biology and microenvironmental work. Crucially, they confront the constraints unique to rare malignancies: small cohorts, batch effects, limited matched multi-omics datasets, and the difficulty of converting complex multi-gene signatures into clinically practical assays.

Taken together, these cancer-focused studies map onto a common workflow—prioritization by AI, validation by traditional experimentation, and re-anchoring of both within the biology of each tumor type. They also expose recurring constraints: limited generalizability across centers, the persistent gap between *in silico* drug-response prediction and clinically actionable prescribing, and the need for prospective, protocolized evaluation of AI-derived biomarkers before they can enter standard-of-care decision-making.

## 3 Beyond oncology: chronic diseases and systems-level pharmacology

While cancer dominates the Research Topic, several contributions extend the Research Topic into chronic non-communicable diseases, where the biomarker–target problem is in some respects even more challenging. Etiological heterogeneity, slow disease trajectories, and the entanglement of pathophysiology with lifestyle exposures all complicate biomarker discovery in this space, and the contributing studies illustrate distinct strategies for confronting these problems.

Luo et al. examine cardiovascular disease (CVD) from a novel angle, reframing nutrition as a source of modifiable molecular signals rather than as a behavioral exposure. By applying AI-driven network pharmacology and integrative omics analyses to nutrition-modulated pathways, the authors map links between nutrient-derived metabolites and established CVD drug targets, surfacing repurposing opportunities and refining target context for known agents. Their treatment of sterol regulatory element-binding proteins and other nutrient-responsive nodes is a useful example of how AI can connect traditionally separate fields—nutritional science, lipid metabolism, and cardiovascular pharmacology—into a single mechanistic framework for biomarker discovery.

Chen et al. tackle early-stage chronic kidney disease (CKD), a condition in which conventional clinical biomarkers—eGFR and albuminuria—are valuable but insufficient for capturing the molecular heterogeneity that drives progression and treatment response. Their review traces the trajectory from single conventional markers to integrative multi-omics signatures, drawing on genomic, transcriptomic, proteomic, and metabolomic studies to argue that combining complementary molecular layers refines prognostic models for CKD trajectories and reveals candidate therapeutic targets that are not apparent from any single-omics analysis. The authors also confront implementation: pragmatic studies and health-economic analyses are needed to determine how multi-omics testing can be integrated with electronic health records and decision-support systems in a cost-effective and equitable manner. This concern—how to translate multi-omics promise into routine clinical workflows—recurs across the Research Topic and is, in our view, the most under-addressed problem in current AI-enabled biomarker research.

Beyond these specific contributions, a wider set of articles in the Research Topic addresses additional disease contexts and methodological themes, including the use of network and systems-pharmacology approaches, AI-assisted drug repurposing, and integration of single-cell and spatial data with bulk-omics signatures. Collectively, the non-cancer contributions reinforce a point that emerges from the oncology articles as well: the value of AI in pharmacology is realized most fully when computational prioritization is tightly coupled with disease-specific mechanistic biology and with the operational realities of the clinic.

## 4 Cross-cutting themes

Three cross-cutting themes emerge across the contributions and warrant explicit articulation.

First, integration is operational, not rhetorical. The contributing studies repeatedly frame integration as an iterative loop in which AI-derived candidates are functionally validated, and in which experimental observations refine the next round of computational modeling. This stands in contrast to many earlier studies that have treated AI and experimental biology as parallel rather than coupled. The osteosarcoma framework articulated by Gao and Wu, the multi-layer thyroid-cancer workflow of Wan et al., and the AFP/PIVKA-II validation logic of Wu et al. all instantiate this iterative model in concrete and reproducible terms.

Second, from single markers to composite signatures. Across tumor types and chronic diseases, the contributors converge on multi-feature panels rather than isolated biomarkers. This shift reflects both the biology—where disease states almost always involve coordinated dysregulation across multiple layers—and the comparative advantage of AI methods, which thrive on high-dimensional patterns. The implication for the field is significant: routine adoption of composite biomarkers will require standardized assays, transparent reporting of model architecture and training data, and pragmatic strategies to compress complex signatures into clinically feasible tests.

Third, translational validation remains the rate-limiting step. Almost every contribution acknowledges, explicitly or implicitly, that the dominant barrier between AI-derived biomarkers and clinical impact is not algorithmic but translational. Limited cohort sizes, batch effects, inter-center variability, weak benchmarking, and the absence of standardized pipelines for evaluating AI biomarkers in real-world workflows all conspire to slow translation. The community-level response will need to include rigorous reporting frameworks (such as DECIDE-AI, SPIRIT-AI, and CONSORT-AI), prospective protocolization of AI-enabled tools, and closer integration of computational scientists, laboratory medicine specialists, and clinicians from the earliest stages of biomarker development.

## 5 Outlook

Looking forward, three priorities follow from the work assembled in this Research Topic. First, generalizable, multi-cohort datasets—particularly in less-studied diseases and underrepresented populations—are essential to test whether AI-derived biomarkers are robust beyond their training environments. Second, mechanistic interpretability must continue to be a first-class objective alongside predictive performance; biomarkers that cannot be linked back to plausible biology will struggle to attract clinical and regulatory confidence. Third, the field must move past demonstration projects toward implementation science: cost-effectiveness analyses, health-system integration studies, and pragmatic trials of AI-enabled biomarker-guided care.

We are grateful to the authors for their thoughtful contributions, to the reviewers whose careful evaluations have shaped the quality of this Research Topic, and to the editorial staff at Frontiers in Pharmacology for their support throughout the development of this Research Topic. We hope that the studies gathered here will both inform and challenge readers, and that they will help accelerate the integration of traditional pharmacological rigor with the analytical reach of artificial intelligence.
